# Research trends and collaboration patterns in robotic retroperitoneal lymph node dissection for testicular cancer

**DOI:** 10.1007/s11701-026-03603-2

**Published:** 2026-06-22

**Authors:** Demirhan Örsan Demir, Çağrı Öktem, Yusuf Gökkurt, Arif Bedirhan Bayraktar, Ahmet Varan

**Affiliations:** 1 Department of Urology, Ankara Bilkent City Hospital, Ankara, Turkey; 2 Department of Urology, Kulu State Hospital, Konya, Turkey; 3 Department of Urology, Ankara Training and Research Hospital, Ankara, Turkey; 4 Department of Urology, Haymana State Hospital, Ankara, Turkey

**Keywords:** Robotic retroperitoneal lymph node dissection, Testicular cancer, Minimally invasive surgery, Robotic surgery, Bibliometric analysis

## Abstract

Robotic retroperitoneal lymph node dissection (RPLND) has become an increasingly used minimally invasive approach in the treatment of testicular cancer. However, the publication trends and scientific structure of the literature in this field have not been fully evaluated. Therefore, this study aimed to analyze the global literature on robotic RPLND in testicular cancer using bibliometric methods. A total of 93 publications published between 2006 and 2025 were included in the analysis. Bibliometric analyses were performed using Biblioshiny and VOSviewer. Publication trends, citation data, leading journals and institutions, international collaborations, co-authorship networks, keyword co-occurrence, and bibliographic coupling patterns were examined. Annual publication output generally increased over time, with the highest number of publications reported in 2021 and 2025 (*n* = 13 each). *Journal of Robotic Surgery* was the most productive journal with 13 publications, followed by *World Journal of Urology* with 12 publications. Johns Hopkins University was identified as the leading institution. The United States ranked first in terms of publication number, citation impact, and international collaboration. Keyword analysis showed that the literature mainly focused on robotic surgery, minimally invasive surgery, post-chemotherapy disease, and nonseminomatous germ cell tumors. In conclusion, publications related to robotic RPLND in testicular cancer have increased over time. The literature is largely generated by a limited number of specialized centers and mainly focuses on minimally invasive surgical approaches and perioperative outcomes. This bibliometric analysis provides an overview of publication trends, collaboration patterns, and major research themes within the robotic RPLND literature.

**Supplementary Information:**

The online version contains supplementary material available at 10.1007/s11701-026-03603-2.

## Introduction

Testicular cancer is one of the most common solid malignancies in young men, and retroperitoneal lymph node dissection (RPLND) plays an important role in the treatment of selected patients with germ cell tumors. Open RPLND has long been accepted as the standard surgical approach because of the technical difficulty of retroperitoneal surgery and the close relationship between lymphatic tissue and major vascular structures [1, 2]. In recent years, however, advances in minimally invasive surgery and robotic technology have increased interest in robotic-assisted RPLND as an alternative approach. The first robotic-assisted RPLND was reported in 2006 and demonstrated the technical feasibility and safety of the robotic platform in testicular cancer surgery [3].

Although robotic RPLND has become increasingly popular, the procedure remains technically demanding and is mostly performed in experienced high-volume centers [1]. Current evidence mainly consists of retrospective studies evaluating perioperative safety, functional outcomes, and short-term oncologic results. Previous studies, including recent comparative analyses and meta-analyses, have suggested that robotic RPLND may offer several perioperative advantages over open surgery, including improved visualization, enhanced surgical dexterity, reduced blood loss, lower transfusion rates, shorter hospital stay, and faster postoperative recovery while maintaining comparable complication and oncologic outcomes [2, 3]. In addition, the available literature is heterogeneous regarding patient selection, surgical indications, operative techniques, and reported outcomes [1]. However, despite the growing number of publications, the overall scientific landscape, collaboration patterns, influential studies, and evolving research topics within robotic RPLND literature have not yet been comprehensively evaluated using bibliometric methods.

Therefore, the aim of the present study was to perform a bibliometric analysis of the global scientific literature on robotic RPLND in testicular cancer. We evaluated publication trends, citation patterns, leading journals and institutions, international collaborations, co-authorship networks, and major research themes in order to better understand the development and current structure of the field.

## Materials and methods

The present bibliometric analysis utilized the Web of Science Core Collection (WoSCC) as the main source for identifying and retrieving relevant publications. WoSCC was selected because it provides structured bibliographic and citation data that are commonly used in bibliometric analyses. A Topic Search (TS) was conducted using the following search strategy: TS = ((robot* OR “robot-assisted” OR “robotic-assisted” OR “robotic surgery” OR “robotic approach”) AND (“retroperitoneal lymph node dissection” OR RPLND OR “retroperitoneal lymphadenectomy”) AND (“testicular cancer” OR “testicular tumor*” OR “testicular tumour*” OR “testicular neoplasm*” OR “germ cell tumor*” OR “germ cell tumour*” OR TGCT OR NSGCT OR SGCT OR seminoma* OR nonseminoma*)). All records were collected on May 13, 2026.

**Fig. 1:**
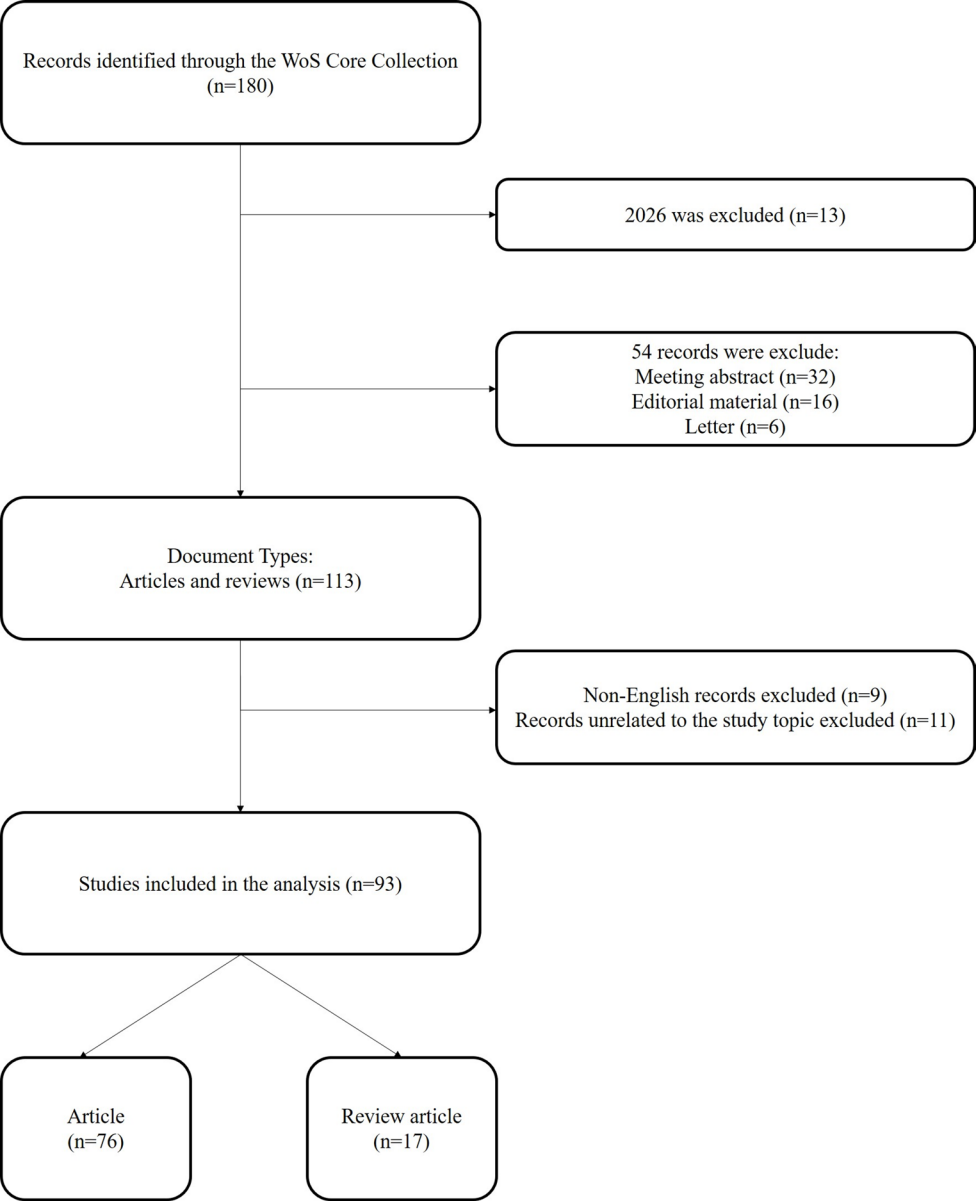
Flow diagram illustrating the literature screening and study selection process used for the bibliometric analysis

The initial search yielded 180 records. Publications from 2026 were excluded because the year was incomplete at the time of data collection, resulting in the removal of 13 records. Following document type filtering, 113 studies categorized as articles or reviews remained. Subsequently, 9 non-English publications and 11 studies unrelated to the research topic were excluded after manual screening. Ultimately, 93 publications were included in the final analysis, consisting of 76 articles and 17 review papers (Fig. 1).

Study selection and screening were performed independently by two investigators. Differences in opinion were resolved through discussion, and a third reviewer was involved when agreement could not be reached.

Bibliometric analyses were conducted using Biblioshiny (version 4.1.3), the web-based interface of the Bibliometrix package in RStudio, together with VOSviewer (version 1.6.20) [4–6].

Annual scientific productivity was evaluated according to the number of publications produced each year. Citation performance was assessed using the average annual citation rate to reduce the influence of publication year differences. In addition, Reference Publication Year Spectroscopy (RPYS) analysis was used to identify influential reference periods within the literature.

Journal productivity was assessed according to the number of published studies, and source growth over time was analyzed to demonstrate publication trends among leading journals. Institutional productivity was evaluated based on the number of publications affiliated with each institution.

Country-based analyses included total citation counts, international collaboration patterns, and corresponding author productivity. Collaboration patterns between countries were visualized using a global collaboration map, while corresponding author analyses distinguished between single-country publications (SCP) and multiple-country publications (MCP).

A co-authorship network analysis was performed to examine collaborative relationships between authors. In this visualization, node size represented author productivity, whereas links between nodes reflected the strength of collaboration. Authors with at least one publication were included in the analysis without applying a citation threshold.

Network analyses, including co-authorship, keyword co-occurrence, and bibliographic coupling, were performed using VOSviewer. Analyses were conducted with the full counting method, and association strength was used as the normalization technique. Within the generated maps, nodes represented individual items and links represented relationships between them. Network connectivity was quantified using Total Link Strength (TLS).

To explore the conceptual structure of the field, a keyword co-occurrence analysis based on author keywords was conducted. Keywords were represented as nodes, and links indicated the frequency with which terms appeared together within the same publications. Node size reflected keyword frequency, and all keywords with at least one occurrence were included.

Bibliographic coupling analysis was additionally performed to identify studies with similar citation patterns and to reveal research groupings within the field. In these visualizations, each node represented a publication, node size reflected citation impact, and links were established according to shared references. Different colors indicated clusters of publications with comparable citation characteristics.

Before analysis, the dataset underwent a standardization process to improve consistency and reduce duplication. Variations in author names and keyword terminology were harmonized using a VOSviewer thesaurus file (Supplementary Materials 1 and 2), thereby improving the reliability and interpretability of the network visualizations.

## Results

**Fig. 2:**
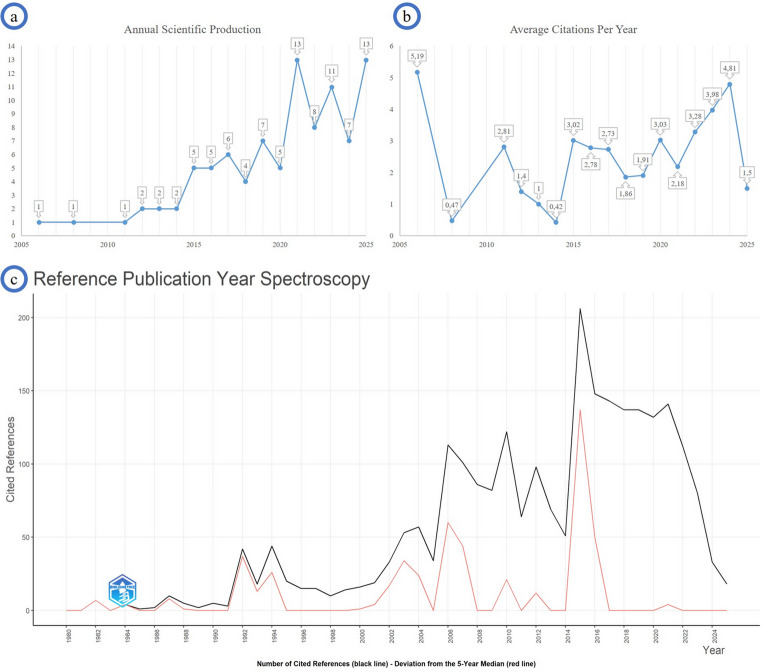
(**a**) Annual publication output of the included studies. (**b**) Mean annual citation rates. (**c**) Reference Publication Year Spectroscopy (RPYS) analysis demonstrating the temporal distribution of cited references and major citation peaks over time

Annual scientific production showed an overall increasing trend over time, with the highest number of publications recorded in 2021 and 2025 (*n* = 13 each) (Fig. 2a).

Average citations per year fluctuated throughout the study period and reached its highest value in 2006 (5.19), followed by 2024 (4.81) (Fig. 2b).

RPYS analysis demonstrated several citation peaks across different years, with the most prominent peak observed in 2015, indicating the presence of highly influential references during that period (Fig. 2c).

**Fig. 3:**
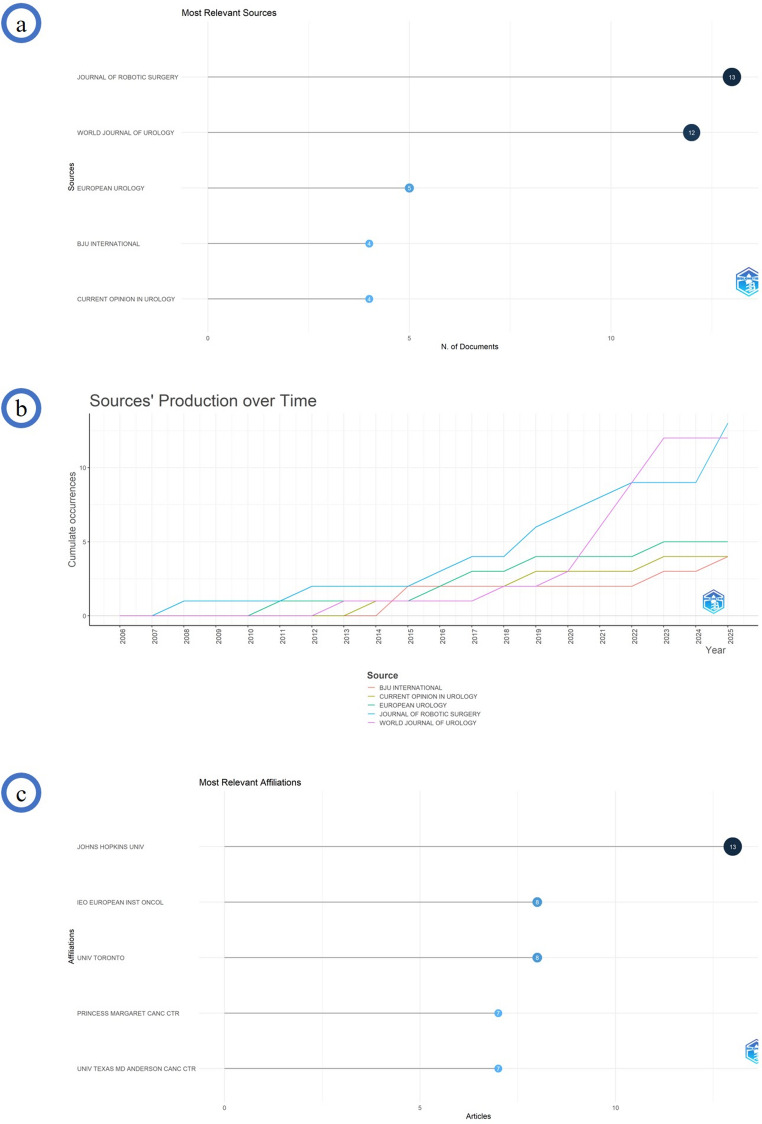
(**a**) Most productive sources based on publication count. (**b**) Temporal trends in publication output among the leading sources. (**c**) Most productive affiliations based on publication count

*Journal of Robotic Surgery* was the leading journal with 13 publications, followed closely by *World Journal of Urology* with 12 publications (Fig. 3a). *European Urology* ranked third with 5 publications, while *BJU International* and *Current Opinion in Urology* each contributed 4 publications. *Journal of Robotic Surgery* demonstrated a marked increase in publication output after 2018 and became the leading source over time. Similarly, *World Journal of Urology* showed rapid growth between 2020 and 2023, reaching 12 cumulative publications by the end of the study period (Fig. 3b). *Johns Hopkins University* was identified as the most productive affiliation with 13 publications (Fig. 3c).

**Fig. 4:**
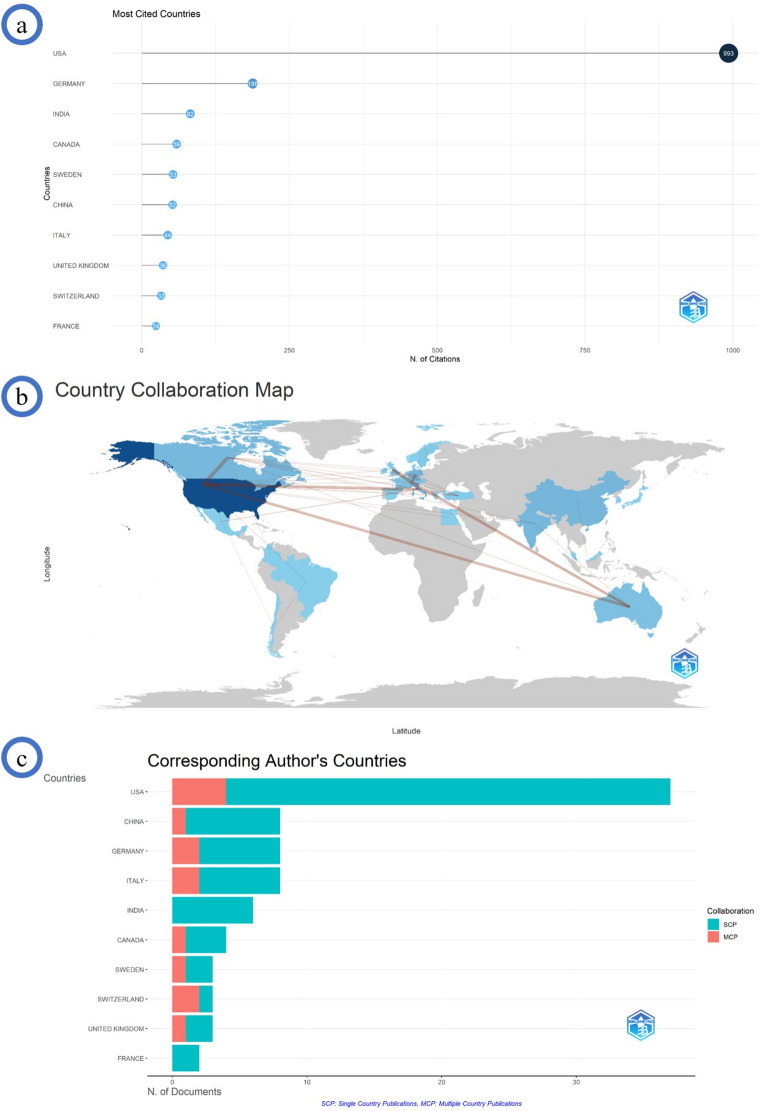
(**a**) Countries with the highest citation counts within the included literature. (**b**) Global collaboration network illustrating international research partnerships between countries. (**c**) Distribution of corresponding author’s countries according to single-country publications (SCP) and multiple-country publications (MCP)

The United States (USA) ranked first in citation impact with 993 citations, followed by Germany (188) and India (82) (Fig. 4a). USA ranked first with 37 articles (39.8%), including 33 SCP and 4 MCP publications. China contributed 8 articles (8.6%) with 7 SCP and 1 MCP publications, whereas Germany and Italy each produced 8 articles (8.6%), including 6 SCP and 2 MCP publications. India ranked fifth with 6 articles (6.5%), all of which were SCP publications (Fig. 4b). The country collaboration map demonstrated that the USA was the central hub of international collaboration, showing the strongest collaborative links with Canada (*n* = 3), Australia (*n* = 2), and Italy (*n* = 2). Additional collaborations were identified between Germany and Switzerland (*n* = 2), Australia and Switzerland (*n* = 2), and the United Kingdom and Switzerland (*n* = 2) (Fig. 4c).

**Fig. 5:**
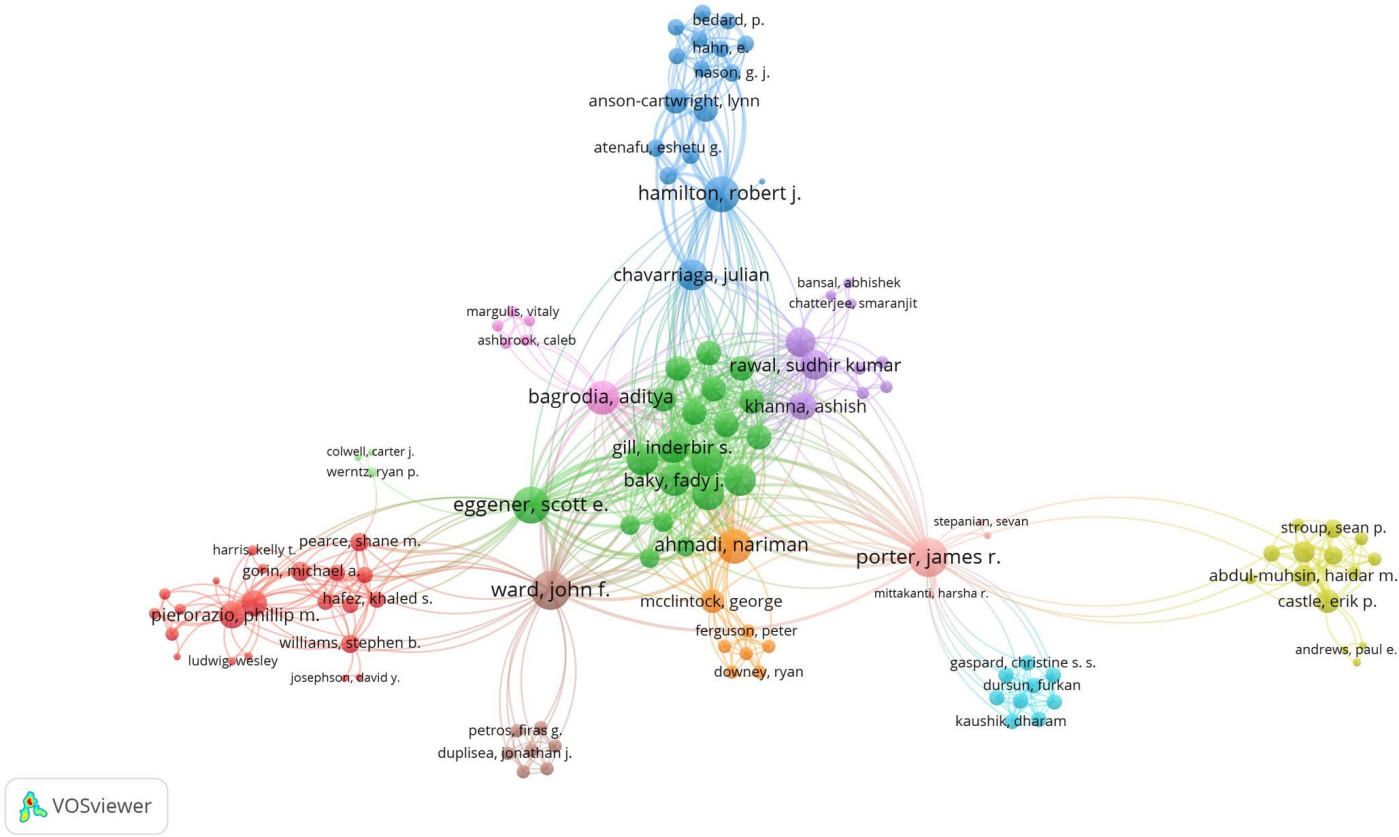
Author-level co-authorship network demonstrating collaborative relationships among the most productive researchers in the field

The author co-authorship network is presented in Fig. 5. Among the 121 authors identified, the network consisted of 11 clusters, 752 links, and a TLS of 864. James R. Porter and John F. Ward had the highest TLS values (57 each), followed by Scott E. Eggener (TLS = 52), Robert J. Hamilton (TLS = 49), and Nariman Ahmadi (TLS = 46). James R. Porter also ranked first in publication count with 7 documents.

**Fig. 6:**
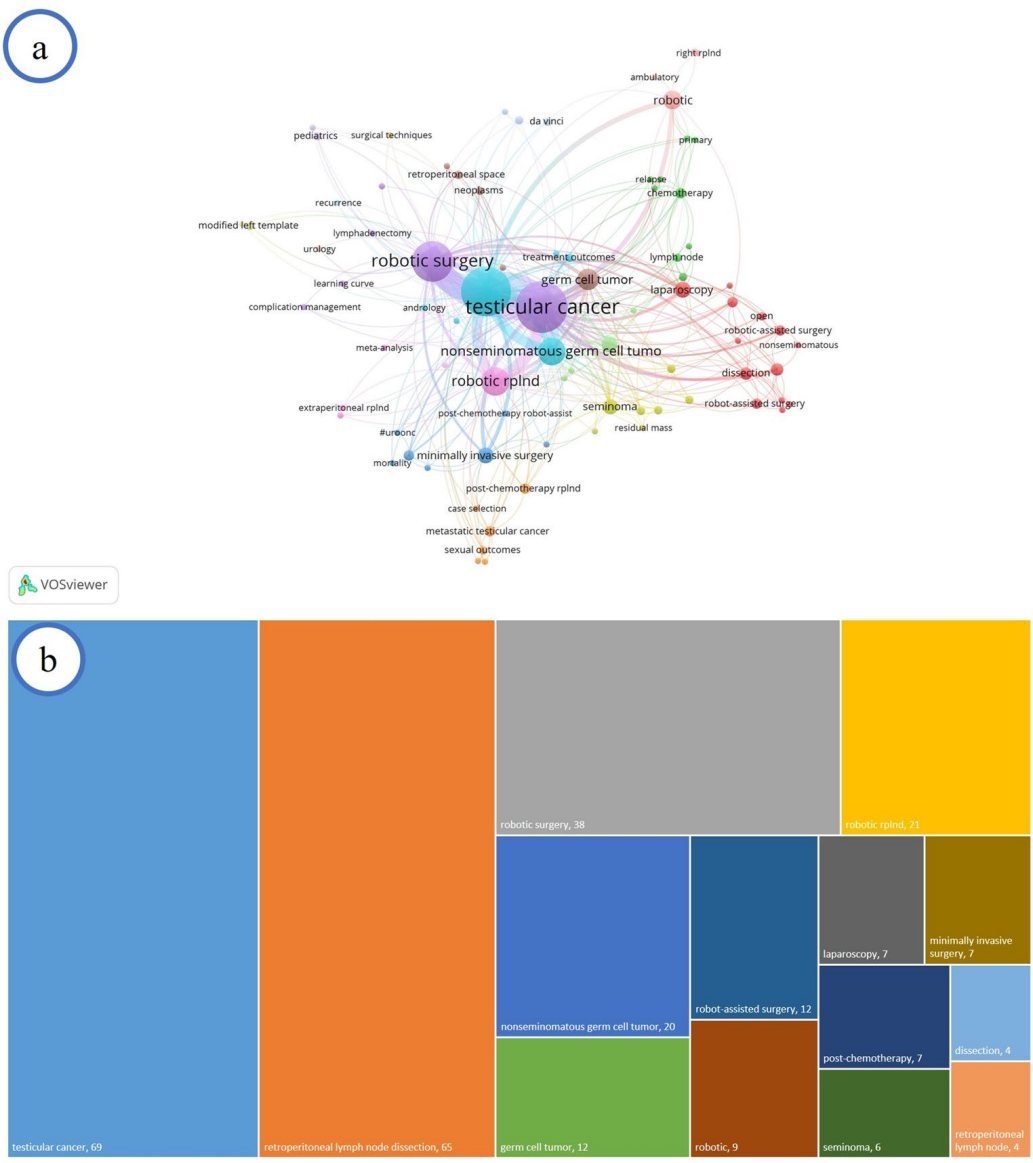
(**a**) Keyword co-occurrence network illustrating the conceptual structure and major research themes within the literature. (**b**) Treemap visualization of the most frequently occurring keywords with a minimum occurrence threshold of 4

Keyword co-occurrence analysis of authors’ keywords identified 81 keywords distributed across 18 clusters, with 349 links and a TLS of 686 (Fig. 6a). The most frequent keywords with the highest TLS values were testicular cancer (69 occurrences, TLS = 241), retroperitoneal lymph node dissection (65 occurrences, TLS = 221), robotic surgery (38 occurrences, TLS = 127), robotic RPLND (21 occurrences, TLS = 78), and nonseminomatous germ cell tumor (20 occurrences, TLS = 75). Authors’ keywords with a minimum occurrence of four are presented using a treemap visualization in Fig. 6b.

**Fig. 7:**
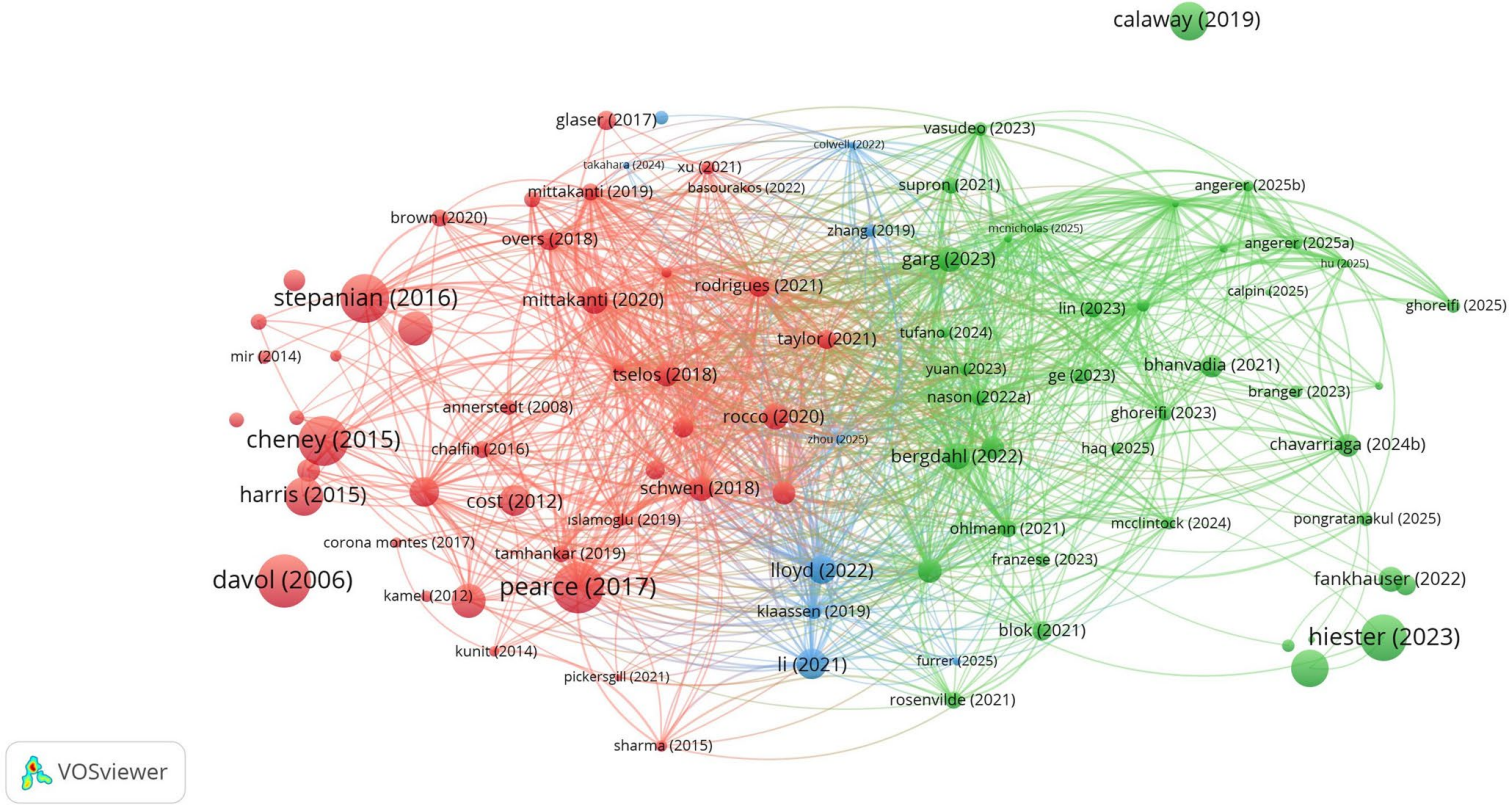
Bibliographic coupling network demonstrating relationships among documents with shared citation patterns within the literature

The bibliographic coupling network is presented in Fig. 7. The analysis identified 93 interconnected documents distributed across 3 clusters, with 3,613 links and a TLS of 13,639. The most highly cited documents were Davol et al. (2006) (109 citations, TLS = 54), Pearce et al. (2017) (97 citations, TLS = 409), and Cheney et al. (2015) (93 citations, TLS = 258) [3, 7, 8].

## Discussion

This bibliometric study provides an overview of the global scientific literature on robotic RPLND in testicular cancer. The findings demonstrated a gradual increase in publication activity over time, particularly after 2014, together with a concentration of research output within a relatively limited number of specialized journals and academic centers. USA was identified as the leading contributor in terms of publication volume, citation impact, and international collaboration. Overall, these findings suggest that robotic RPLND has become an increasingly investigated topic within minimally invasive urologic oncology.

The increase in publication activity observed during the last decade may reflect the growing interest in robotic applications for complex retroperitoneal surgery. RPLND remains a technically demanding procedure because of the challenging retroperitoneal anatomy and the close relationship between lymphatic tissue and major vascular structures. The robotic platform may provide potential technical advantages, including enhanced visualization and improved dexterity during dissection. Early multicenter experiences demonstrated that robotic RPLND could be performed with acceptable perioperative morbidity and encouraging short-term oncologic outcomes in selected patients with low-stage nonseminomatous germ cell tumors [7]. Subsequent reports also supported the feasibility of robotic approaches in both primary and post-chemotherapy settings, particularly in experienced referral centers [9, 10]. Recent comparative studies have also reported lower blood loss and shorter hospital stay with robotic RPLND compared with open RPLND, while oncologic outcomes appear largely comparable in appropriately selected patients [11]. Comparable findings have been reported in other robotic urological procedures, including robot-assisted radical prostatectomy and robot-assisted partial nephrectomy, where robotic approaches have been associated with favorable perioperative outcomes and, in selected settings, improved surgical quality metrics compared with conventional techniques [12, 13].

*Journal of Robotic Surgery* and *World Journal of Urology* were identified as the leading publication sources, while *Johns Hopkins University* emerged as the most productive institution [14, 15]. USA occupied a central position in both citation and collaboration analyses, likely reflecting earlier access to robotic technology and the presence of specialized multidisciplinary oncologic programs. In addition, the collaboration network suggested that scientific interaction was mainly established between centers in North America, Europe, and Australia [10, 16].

The co-authorship analysis demonstrated that robotic RPLND research is shaped by a relatively limited but interconnected group of researchers, largely originating from experienced high-volume centers. The keyword co-occurrence analysis provided additional insight into the evolving research interests within the field. Alongside procedural terminology, the network also highlighted increasing attention toward topics such as post-chemotherapy disease, residual masses, seminoma, and minimally invasive surgery. The close relationship between these terms suggests that the literature has gradually expanded beyond initial feasibility-focused reports toward more clinically complex scenarios and broader discussions regarding patient selection and surgical indications. In particular, the growing visibility of terms associated with post-chemotherapy RPLND may reflect increasing interest in evaluating robotic approaches in technically challenging oncologic settings [9, 17]. Overall, these findings suggest that contemporary robotic RPLND research is evolving toward more comprehensive evaluation of perioperative and oncologic outcomes. From a clinical perspective, the increasing focus on topics such as post-chemotherapy disease and minimally invasive surgery suggests growing interest in defining the role of robotic RPLND in more complex clinical settings. The identified research trends may also help guide future studies toward long-term oncologic, functional, and quality-of-life outcomes, which remain important areas of investigation.

Several limitations should be considered when interpreting the findings of this study. The analysis was limited to publications indexed in the WoSCC and written in English, which may have resulted in exclusion of relevant studies from other databases or languages. Although the use of a single database enabled a consistent and standardized approach to bibliographic and citation analyses, reliance on WoSCC alone may have introduced database selection bias and may not have captured all relevant publications indexed in databases such as Scopus, PubMed/MEDLINE, or Embase. In addition, citation-based analyses may inherently favor older publications. Nevertheless, this study represents one of the first bibliometric evaluations specifically focused on robotic RPLND in testicular cancer and provides a comprehensive overview of publication trends, collaborative structures, and major research themes within the field.

In conclusion, publications related to robotic RPLND in testicular cancer have increased over time, particularly during the last decade. The literature is largely generated by a limited number of specialized centers and mainly focuses on minimally invasive surgical approaches and perioperative outcomes. The present bibliometric analysis summarizes publication trends, collaborative networks, and research themes within the field and may serve as a reference for future research in robotic RPLND.

## Supplementary Information

Below is the link to the electronic supplementary material.

Supplementary Material 2

Supplementary Material 1

## Data Availability

The datasets analyzed during the current study are available from the corresponding author upon reasonable request.
